# *In vivo* hamster cheek pouch subepithelial ablation, biomaterial injection, and localization: pilot study

**DOI:** 10.1117/1.JBO.27.8.080501

**Published:** 2022-08-25

**Authors:** Ilan Gabay, Kaushik Subramanian, Liam Andrus, Andrew DuPlissis, Murat Yildirim, Adela Ben-Yakar

**Affiliations:** a University of Texas at Austin, Department of Mechanical Engineering, Austin, Texas, United States; b University of Texas at Austin, Department of Biomedical Engineering, Austin, Texas, United States

**Keywords:** ablation of tissue, ultrafast lasers, nonlinear microscopy, *in vivo*, vocal fold scarring, hamster cheek pouch

## Abstract

**Significance:**

The creation of subepithelial voids within scarred vocal folds via ultrafast laser ablation may help in localization of injectable biomaterials toward a clinically viable therapy for vocal fold scarring.

**Aim:**

We aim to prove that subepithelial voids can be created in a live animal model and that the ablation process does not engender additional scar formation. We demonstrate localization and long-term retention of an injectable biomaterial within subepithelial voids.

**Approach:**

A benchtop nonlinear microscope was used to create subepithelial voids within healthy and scarred cheek pouches of four Syrian hamsters. A model biomaterial, polyethylene glycol tagged with rhodamine dye, was then injected into these voids using a custom injection setup. Follow-up imaging studies at 1- and 2-week time points were performed using the same benchtop nonlinear microscope. Subsequent histology assessed void morphology and biomaterial retention.

**Results:**

Focused ultrashort pulses can be used to create large subepithelial voids *in vivo*. Our analysis suggests that the ablation process does not introduce any scar formation. Moreover, these studies indicate localization, and, more importantly, long-term retention of the model biomaterial injected into these voids. Both nonlinear microscopy and histological examination indicate the presence of biomaterial-filled voids in healthy and scarred cheek pouches 2 weeks postoperation.

**Conclusions:**

We successfully demonstrated subepithelial void formation, biomaterial injection, and biomaterial retention in a live animal model. This pilot study is an important step toward clinical acceptance of a new type of therapy for vocal fold scarring. Future long-term studies on large animals will utilize a miniaturized surgical probe to further assess the clinical viability of such a therapy.

## 1 Introduction

Vocal fold (VF) scarring is one of the predominant causes of voice disorders, affecting an estimated 3 to 10 million people in the United States alone.^1^^,^^2^ VF scarring results from inflammation or injury of the mucosa, leading to the formation of scar tissue within a subepithelial tissue layer, the lamina propria (LP). Scar tissue within the LP increases tissue stiffness and degrades, or, in severe cases, eliminates voice function.^3^ Histologically, scar formation within the LP is associated with an increased density and uniformity of thick collagen fibers and decreased density of elastin fibers and hyaluronic acid.^4^ Although treatment of VF scarring remains a challenge, one promising treatment involves injection of therapeutic biomaterials into the scarred LP to restore VF viscoelasticity.^3^ Over the past two decades, many biomaterial and tissue engineering approaches have been investigated to repair scarred VF, including hydrogels,^5^^,^^6^ stem cells,^7^^,^^8^ hepatocyte growth factor,^9^^,^^10^ and basic fibroblast growth factor.^11^ In a previous *in vivo* study, Karajanagi et al. injected a polyethylene glycol-based hydrogel (PEG30) into healthy canine VF to determine its effects on tissue morphology and vocal performance.^12^ The canine subjects were endoscopically screened for disorders over 4 months wherein injection of PEG30 showed no adverse effects on mechanical stability or voice function. It was concluded that PEG30 exhibited biocompatibility with minor side effects and showed promise for treatment of human VF scarring. Although these results were encouraging, injection of soft biomaterials into scarred VF is likely to be challenging due to the presence of dense scar tissue.^13^^,^^14^ Indeed, injection-based delivery techniques have been shown to suffer from poor repeatability due to the difficulty in localizing the injected material in the desired plane within the LP.^12^^,^^15^^–^^17^ For example, studies by Hoy et al. indicated that injection into scarred hamster cheek pouches led to significant backflow of the biomaterial along the point of injection, with very little volume retained within the scar tissue.^17^ One possible solution to address this challenge could be to create subepithelial voids in scarred VF to provide an injection space for these biomaterials, aiding their localization at the desired depth.

The ultrafast laser ablation process relies on rapid nonlinear absorption at the focal plane, confining energy deposition within the focal volume and resulting in minimal thermal damage to surrounding tissues.^18^^,^^19^ Such a high degree of spatial and thermal confinement enables precise material removal inside bulk tissues, which is especially important when working with delicate biological systems such as the larynx. Successful laser microsurgery of VF using ultrashort pulses has been previously demonstrated by our group; it showed the potential for subsurface ablation confined within the sublayers of the LP without creating any adjacent tissue damage.^17^^,^^20^^–^^23^ In these studies, high numerical aperture focusing optics were employed to reduce the pulse energies required for ablation. In addition, pulse widths in the picosecond (ps) range limited the peak pulse power propagating through the tissue. Together, laser surgery with tightly focused beams and ps-pulses can negate out-of-focus damage caused by self-focusing-induced focal plane shifting to confine ablation to a subsurface plane.^24^ Focused ultrashort laser pulses may also be used to visualize intrinsic molecular and morphological tissue properties in the surgery site through nonlinear optical microscopy. Two-photon fluorescence (TPF) and second harmonic generation (SHG) microscopy can provide complementary information on the structure and composition of the cells and collagen composing VF and can thus provide image guidance for ultrafast laser microsurgery. Toward clinically viable ultrafast laser surgery, our group has previously developed several microsurgery probes with and without image-guidance and continues to design probes.^25^^–^^29^

In this letter, we demonstrate a proof of concept for the creation of subepithelial voids, injection of a biomaterial, and retention of the biomaterial inside a void using an *in vivo* animal model and a benchtop microscope. We used hamster cheek pouches, which are considered a good model for VF surgery because their epithelium and LP layers are very similar in composition to humans.^20^ In addition, hamster cheek pouches are easily accessible using a benchtop system.

## 2 Methods

A polyethylene glycol-based hydrogel (PEG30) was prepared by our collaborators at Massachusetts General Hospital’s Department of Laryngeal Surgery and Voice Rehabilitation. The hydrogel’s biocompatibility, mechanical functionality, and retention were shown in canine models as part of previous work.^12^ The PEG30 used in our experiments was tagged with fluorescent rhodamine dye to aid in visual identification. Briefly, PEG30 hydrogels were prepared by photopolymerization of a solution containing PEG diacrylate (30% v/v, hence the name PEG30) in the presence of PEG (both with a molecular weight of 10 kDa). A photo-initiator (Irgacure 2959, 0.05% w/v) was added to the solution prior to UV polymerization ($10\;W/{\mathrm{cm}}^{2}$ at 365 nm) for 200 s. After a 24-h incubation period in PBS at 37°C, swollen gels were sheared through progressively smaller bore needles to prepare them for injection.

The animal studies were performed in accordance with the PHS Policy on Humane Care and Use of Laboratory Animals, the NIH Guide for the Care and Use of Laboratory Animals, and Animal Welfare Act. We followed protocols approved by the Institutional Animal Care and Use Committee of the University of Texas at Austin. Four male Syrian hamsters (young adults 80 g wt.) were obtained from Charles River Laboratories and housed at the Animal Resources Center where they were acclimatized for 1 month prior to the experiments. Before each procedure, the hamsters were administered buprenorphine (0.05  mg/kg) and kept under anesthesia throughout the procedure with intraperitoneal ketamine/xylazine (100/7  mg/kg). We used a rectal thermometer and heating pad to regulate body temperature throughout the procedure. Respiratory function and depth of anesthesia were monitored periodically through visual and tactile observation. After surgery, anesthesia was reversed using atipamezole. The hamsters were monitored until they regained consciousness; then they were given buprenorphine (0.05  mg/kg) subcutaneously twice daily for a follow-up period of 2 days. For scarred animal studies, we unilaterally scarred the cheeks of two of the four hamsters (scar group) 1 month prior to surgeries. Specifically, a ∼5-mm circular area was lightly cauterized using electrosurgery (Conmed Saber 2400).

We used a benchtop nonlinear microscope to perform surgery and imaging experiments. The details of this microscope have been previously described.^20^^,^^22^ Briefly, a frequency doubled erbium-doped fiber laser (2 W, 1552  nm/776  nm, Discovery, Raydiance Inc.) facilitated ultrafast laser ablation, TPF imaging, and SHG imaging. Two modes of laser operation were available: a 303 kHz repetition rate with 1.5 ps pulses for surgery and a 2 MHz repetition rate with 1 ps pulses for nonlinear imaging. The laser can be frequency doubled to 776 nm at 36% conversion efficiency to produce maximum pulse energies of 2.4 and 330 nJ at the laser output for 303 kHz and 2 MHz, respectively. Considering the 42% transmission efficiency of our microscope, the laser can provide maximum pulse energies of 1  μJ and 140 nJ at the tissue surface for operation at 303 kHz and 2 MHz, respectively. The laser beam was raster scanned onto the VF tissue by two galvanometric mirrors and expanded to the back aperture of a 20×, 0.75 NA objective (Nikon Plan Apo), providing a $400\times 400\;\mu m^{2}$ field of view (FOV). The objective produced lateral and axial intensity point spread functions with ${1/e}^{2}$ diameters of 1.39±0.16 and 5.2±0.6  μm, respectively. For imaging, emission light was epi-collected, diverted through the appropriate filters in two different collection channels, and detected by photomultiplier tubes (H7422-40, Hamamatsu). Images were generated by averaging 10 frames. The galvanometric mirrors, stage, and PMT were controlled and synchronized using MPScan software.^30^ A simple USB-handheld microscope was used to image the tissue surface with white light for additional guidance during experiments.

**Fig. 1:**
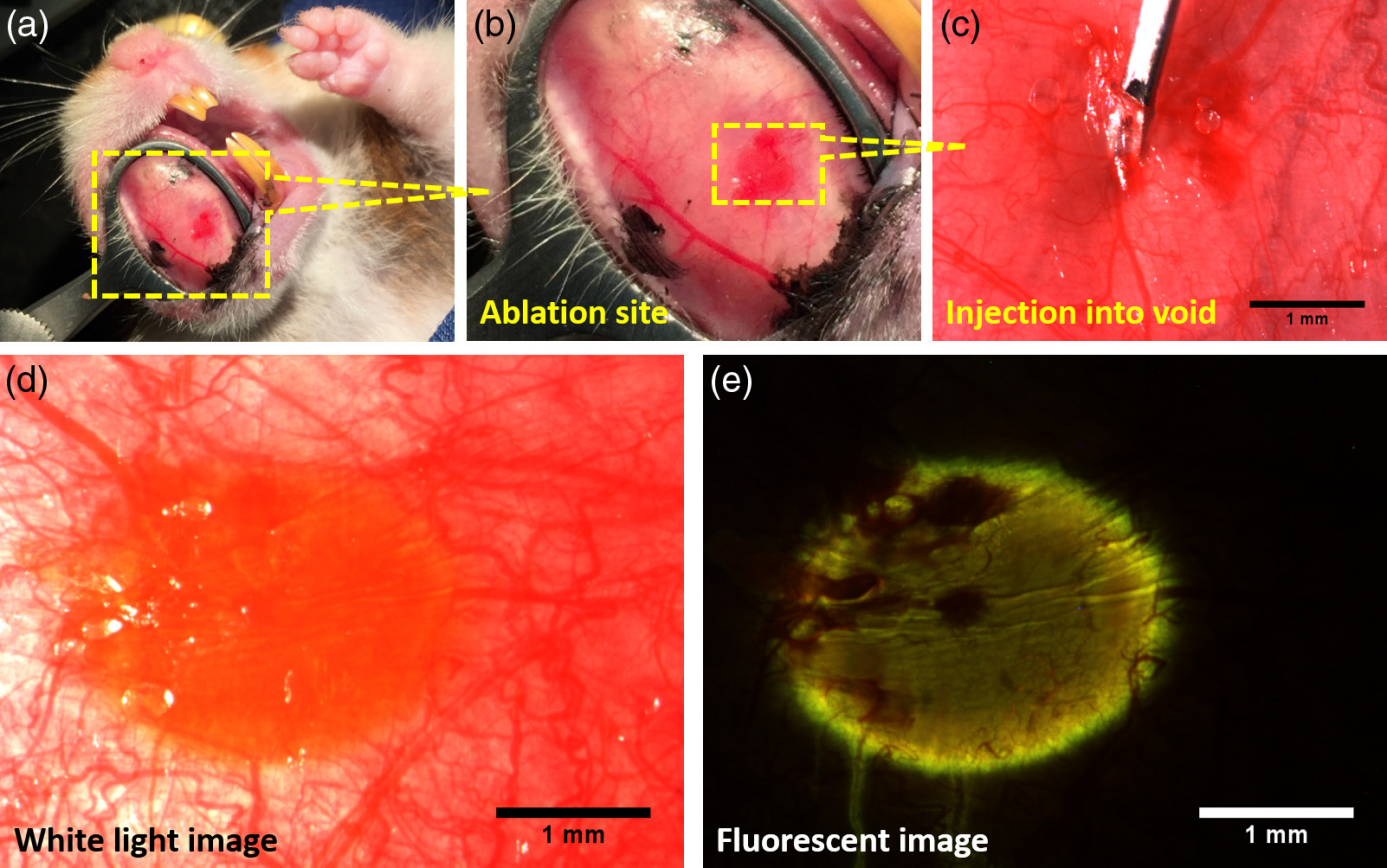
*In vivo* setup for ablation and biomaterial injection in hamster cheek pouch. (a) Hamster cheek pouch everted and clamped with a Desmarres Chalazion clamp. (b) A zoomed-in image showing the black tattoo ink marks, some blood vessels, and the treated area that underwent ablation and is now ready for the biomaterial injection. (c) A 30-gauge needle piercing through the epithelium and aimed toward the ablated void. (d) The rhodamine-tagged PEG30 appears as a red-orange oval under the epithelium, localized inside the void. (e) The same FOV as in (d) with the FITC filter showing the fluorescence of the rhodamine with blood stains appearing as dark red.

Immediately before the image-guided surgery procedure, the hamster was anesthetized, the rectal thermometer was inserted, and the hamster was placed onto the heating pad in a supine position. We everted the cheek pouch of the anesthetized hamster using a modified Desmarres Chalazion clamp, marked the surgery site using black tattoo ink [Figs. 1(a) and 1(b)], and positioned the hamster in our benchtop microscope. Tattooing allowed us to easily identify the region of ablation/injection for follow-up histological analysis. A petri dish was placed on top of the everted cheek pouch to prevent condensation on the microscope objective. Initially, the cheek surface was brought to focus by collecting autofluorescence from the epithelium using TPF imaging and then to a depth at which collagen fibers were first seen below the epithelium.

For the subepithelial ablation procedure, we were interested in getting the most energetic pulses and, therefore, worked at the lower repetition rate of 303 kHz, generating 580 nJ pulses on the tissue surface. Assuming beam attenuation through the ∼43-μm thick epithelium, which can be described using Beer’s law, and taking into account the hamster cheek pouch scattering lengths and fluence thresholds reported by Yildirim et al.,^20^ we calculated the laser fluence at the focal plane right below the epithelium as $∼4.8\;J/{\mathrm{cm}}^{2}$. This fluence value is ∼8× and 12× higher than the measured ablation threshold for scarred and healthy cheek pouches, respectively.^20^ Further, the pulse peak power of ∼0.36  MW is far below the expected critical peak power of ∼3  MW for self-focusing in tissues, suggesting that self-focusing-induced focal plane shifting should not cause any out-of-focus damage during subepithelial ablation.^29^^,^^31^ Given the FOV of $400\times 400\;\mu m^{2}$, laser spot size of 1.39  μm, repetition rate of 303 kHz, and frame rate of 1.11 frames per second, we generated three overlapping pulses on each point inside the FOV.^20^^,^^22^ After ablating the full FOV, the hamster was translated laterally with a small overlap with the neighboring ablation FOV. Overall, we created a 2  mm×400  μm void within the LP right below the epithelium. A similar procedure was carried out on all hamsters; in the scar group, the ablation site was chosen within the scar site.

Immediately following surgery, the hamster was moved to a microinjection setup consisting of a stereoscope (SZX16, Olympus) and a three-axis micromanipulator (KITE-L, World Precision Instruments, LLC). The micromanipulator was used to precisely position a 1-mL syringe with a 30-gauge needle (BD syringe) to inject a small amount of rhodamine-tagged PEG30 into the void [Fig. 1(c)]. The needle was then retracted, and the tissue surface was wiped for any excess material using alcohol pads [Fig. 1(d)]. We then fluorescently visualized the injected dye using the stereoscope’s FITC filter set [Fig. 1(e)].

**Fig. 2:**
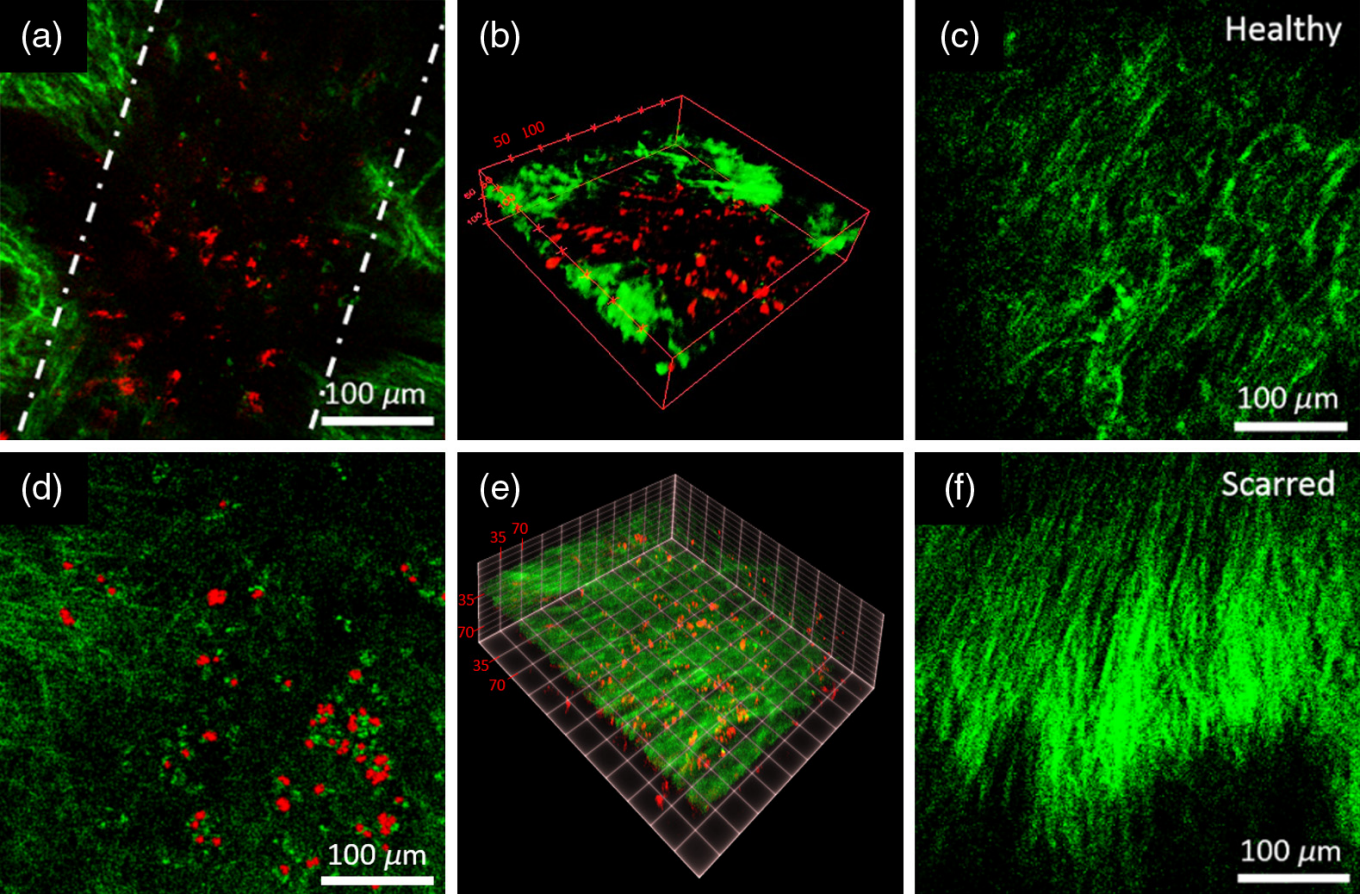
Nonlinear imaging of hamster cheek pouches after surgery and rhodamine-tagged PEG30 injection. (a) A cross-sectional image and (b) a 3D image of the biomaterial-filled void 2 weeks postoperatively in the nonscar group. Rhodamine particles (red) were resolved using TPF imaging and are localized within the subepithelial void. Collagen fibers (green) were resolved using SHG imaging. Dashed lines in (a) show the assumed void margins. The length of the void extends outside the FOV of this figure. The top 25  μm of epithelium was removed in (b) to clearly visualize rhodamine within the subepithelial void. (c) SHG image of collagen fibers in the nonscar group in a region adjacent to the biomaterial-filled void shown in (a) and (b). Fibers appear highly disorganized in (c), as expected for healthy tissue. Collagen fiber structure near the void margins in (a) resembles those shown in (c), confirming that the ablation process does not cause scarring. (d) A cross-sectional image and (e) a 3D image of the biomaterial-filled void 2 weeks postoperatively in the scar group. (f) SHG image of collagen fibers in the scar group. Fiber alignment/organization in (d) indicates presence of scar tissue.

Hamsters were brought back for imaging following a 1- or 2-week recovery period to evaluate biomaterial localization and retention in ablated voids. In both groups of scarred and nonscarred animals, one animal was imaged 1 week after injection, and the other animal was imaged 2 weeks after injection. The ink marks imaged by white light provided a guideline to approximately align the treated area within the FOV of the nonlinear microscope. We performed volumetric TPF imaging and SHG imaging to locate the rhodamine particles and to identify the collagen-rich LP layer containing the void (Fig. 2). The imaging powers that were needed to detect the bright rhodamine particles were not sufficient for also detecting autofluorescence from the epithelium; thus we only show the TPF signal collected from rhodamine overlaid with SHG signal collected from collagen in Fig. 2. In both nonscar and scar groups, overlaid images show the rhodamine-tagged PEG30 localized within the void 2-weeks postoperatively [Figs. 2(a) and 2(d)]. Figures 2(b) and 2(e) show 3D image stacks of the biomaterial-filled void 2 weeks postoperatively in the nonscar and scar groups, respectively. Rhodamine particles were clearly visible inside the voids with an average width of a few hundreds of microns and a length extending beyond the FOV of the images, as would be expected for the 2-mm long ablation area.

## 3 Results and Discussion

Nonlinear imaging of the scar group showed dense and organized collagen fibers, emphasizing the expected difference between scarred tissue in Fig. 2(f) versus a more random orientation of the collagen in Fig. 2(c) for the nonscar samples. In the nonscar group, the collagen fibers around the ablation void in Fig. 2(a) remained disorganized, similar in appearance to those in the nonscarred fibers [Fig. 2(c)] confirming that the ablation process does not introduce any scarring to healthy tissue after 2 weeks. In the case of the scar group, we observed dense and organized collagen fibers throughout the 3D volume [Fig. 2(e)], indicating that subepithelial ablation and biomaterial injection was realized within the scarred cheek pouch.

We imaged the voids of one nonscar and one scar animal 1 week after the surgery and found that the imaging results 1 and 2 weeks postoperatively were similar in the nonscar group. For 1 week follow-up imaging studies of the scar group, however, we were not able to see any rhodamine particles. A possible explanation is suggested following the histology for that animal, which is discussed next.

**Fig. 3:**
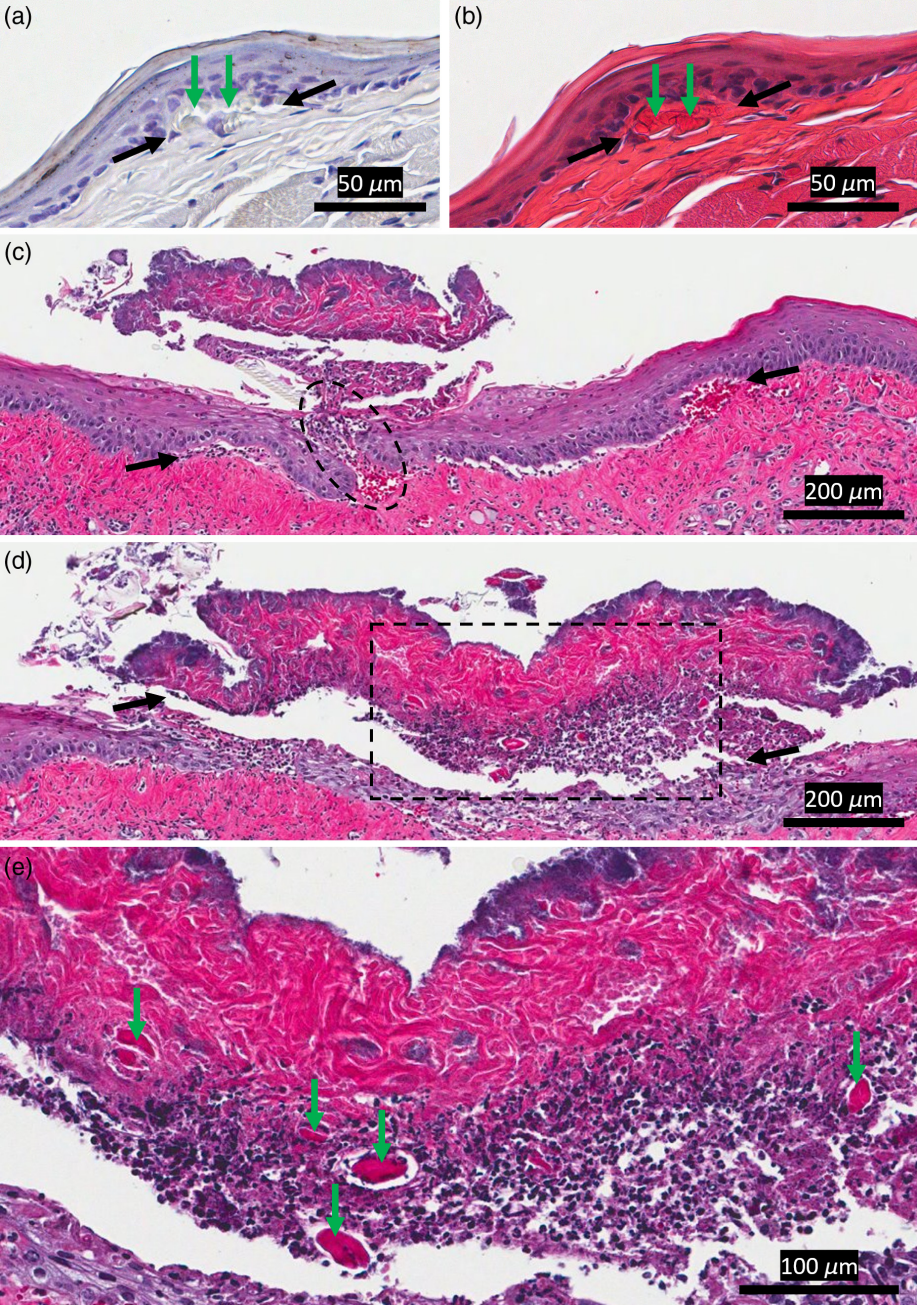
Histology of nonscarred and scarred tissues 2-weeks and 1-week after surgery, respectively. (a) Collagen I antibody staining reveals the void created in the nonscar group 2-weeks postsurgery with (b) H&E staining showing the rhodamine particles. (a) and (b) Consecutive slides (4  μm separation). (c) A scarred tissue shows a collapsed void under the epithelium. The dashed oval is the needle entry location. (d) A slide ∼28  μm away from the slide shown in (c) shows complete separation from the underlying tissue, which may have been caused by fixation and sectioning. The dashed square indicates the FOV shown in (e). (e) A zoomed-in image of the slide shown in (d). Rhodamine particles are seen in the separated tissue layer. Black and green arrows in (a)–(e) indicate void margins and rhodamine particles, respectively.

Immediately after follow-up imaging studies at 1- and 2-week timepoints, animals were sacrificed and prepared for histology. For the nonscar group, histology revealed a similar picture to that of nonlinear microscopy: we were able to detect the void when stained with collagen I antibody [Fig. 3(a)]. A consecutive slide (4  μm away) stained with H&E revealed rhodamine particles inside the void [Fig. 3(b)]. The void was smaller than the size that we created with the laser. One possible reason is that surgery and nonlinear imaging were done while the cheek was stretched, corresponding to smaller dimensions when the tissue was relaxed and fixed in formalin. Histological examination of the scarred tissue with one week follow-up [Fig. 3(c)] showed that the rhodamine-tagged PEG30 might not have localized inside the void for that animal, potentially due to poor healing of the biomaterial injection site (needle entry hole). The slide shown in Fig. 3(c) corresponds to a cross section containing the injection site (dashed oval). Here, we observed an obvious trace of the needle entry hole along with increased epithelial and red blood cell proliferation, indicating possible inflammation and poor healing near the injection site. Incomplete closure of the needle entry hole may have caused PEG30 to leak out of the void over time. Still, a clear gap between the epithelium and LP, which corresponds to the collapsed ablation void, is seen. Alternatively, the absence of PEG30 may be related to tissue damage during preparation for histology and not due to incomplete closure of the injection site. Histology of the 2-week scar group may have also been damaged during tissue preparation, and we were not able to obtain reliable results from this specific sample. The slide in Fig. 3(d) [∼28  μm away from the slide shown in Fig. 3(c)] for the 1-week scar group shows that the tissue above the void was completely separated from the underlying tissue. This separation may have been caused during tissue fixation and/or sectioning, which may have caused the PEG30 to leak out. However, we do observe some residual PEG30 [Fig. 3(e)], indicating partial PEG30 localization in scarred tissue after 1 week.

## 4 Conclusion

In summary, these studies describe our ongoing efforts toward establishing a treatment modality to localize therapeutic biomaterials inside scarred VFs. By enabling precise localization of injectables, this method may be able to help with testing the efficacy of various biomaterials in the treatment of scarred tissue. Further, injection and long-term localization of PEG30 within subepithelial voids in both scarred and nonscarred hamster cheek pouches is an important milestone toward clinical viability of the proposed VF scarring therapy. Retention of PEG30 within these voids over a 2-week period was confirmed both by nonlinear microscopy and histological examination in both scar and nonscar groups. Results for the nonscar group also revealed a disorganized (i.e., healthy) collagen structure at the voids’ margins and suggest that ultrafast laser ablation does not elicit formation of scar tissue.

Although these results were encouraging, clinical translation is limited by the free space delivery of light and the large optics of the benchtop microscope used in these studies. Flexible delivery of tightly focused ultrashort pulses through miniaturized optical systems is required to translate our VF scarring therapy to the clinic. Thus, future studies performed on large animals utilizing miniaturized laser surgery probes with high numerical aperture miniaturized objectives will help to assess the clinical viability of the proposed VF scarring treatment.
